# On the Work of a Military General Hospital in Egypt

**Published:** 1916-07

**Authors:** Ernest Hey Groves

**Affiliations:** Late Major, R.A.M.C., in charge of the Surgical Division of the 21st General Hospital


### ON THE WORK OF A MILITARY GENERAL HOSPITAL IN EGYPT.

BY

ERNEST HEY GROVES, M.S., F.R.C.S.,

*Late Major, R.A.M.C., in charge of the Surgical Division of the 21st General Hospital.*

OF the many thousands of cases which passed through our hands one can speak of only a few, and for the purpose of simplicity I would confine myself to certain groups of cases.

*Enteric Fever.*—At one time we had many hundreds of cases of typhoid and paratyphoid. The most notable general feature about these was the mildness of the disease and the difficulty of diagnosis of the more trivial cases. Surgically there were two main points of interest. The first related to perforations. Of these we had twelve, all of which were operated upon. In only one case was a wrong diagnosis of perforation made, and that was in an officer who was brought in in an almost moribund condition, and about whom no history was obtainable. All our twelve cases were operated upon, but we had only three recoveries. The operations, which varied in severity from mere insertion of a drainage tube into the pelvis or suturing an ulcer up to an excision of the ulcer-bearing segment of the ileus, were generally done under morphia and hyoscine and a local novocain anæsthetic. The other complication which frequently brought the enteric patient to the surgical section was involvement of the gall-bladder. This was observed in three well-marked gradations. In the first an intermittent painful swelling of the gall-bladder occurred. These all did well after drainage, and in every case a culture of the B. typhosus was obtained. Some made spontaneous recoveries without operation, and these perhaps will go to swell the army of "typhoid carriers." In the second grade a persistent tender swelling occurred in the gall-bladder region. On operation there was adhesive cholecystitis, but here again all cases operated upon made a good recovery. In the third type, fortunately the rarest, the gall-bladder actually perforated, and these all proved fatal in spite of operation.

*Dysentery.*—This was indeed the scourge of the Army, and delayed cases arrived in their hundreds; and many of those who died of wounds were found to have the disease in a latent condition, whilst many a man admitted to a surgical ward eventually succumbed to the more insidious disease. Three surgical points only will the limits of this article allow me to touch upon. Appendicostomy from a practical point of view proved itself to be disappointing. This I am convinced was due chiefly to the difficulty in selection of the cases. A very large proportion did well under emetine, and it seemed only right to apply this remedy to every case for a sufficient period to give it a fair trial. At the end of the fortnight occupied by this treatment the cases which remained unrelieved were generally so very ill that no surgical treatment had any chance of success. Perforation of dysenteric ulcers was very rare; in our cases it occurred either in the transverse or sigmoid portions of the colon, and whether operated upon or not, it always proved fatal. The occurrence of hepatic abscess was, as compared with other epidemics before the use of emetine, a very rare complication. In all we had only five cases, three of which recovered and two died. In those cases which did well we were much struck by the way in which the abscess rapidly disappeared, the cavity becoming obliterated, and the operation wound healing within a fortnight. This happy result was no doubt due to early diagnosis, for which we had the invaluable assistance of Sir Ronald Ross, one of the Consulting Physicians to the Forces.

*General Problems of Sepsis and its Treatment.*—The wounded from Gallipoli presented the same deplorable spectacle of severe sepsis which the present war has familiarised us with. The anærobic infections, both tetanus and gas gangrene, were, however, of rare occurrence. Curiously enough, several of our cases of tetanus occurred in association with frost-bites where there had been only the most trivial breach of skin surface. But the ordinary sepsis was rampant, and every lacerated wound gave a culture of either a streptococcus, staphylococcus, bacillus pyocyaneus or bacillus coli. As regards the treatment of severely-infected wounds our experience, so far as it went, did not support the claims set forth by various authorities for the superiority of any one chemical over another.

We used (1) ordinary antiseptics, *e.g.* carbolic and perchloride, (2) peroxide of hydrogen, (3) salt solutions of various strengths, and (4) Eusol. Although each of these methods had its advocates, I think I may fairly say that there was no recognisable difference in the results produced by each. It has to be borne in mind that we dealt only with septic conditions of seven to ten days' standing. The two things which did make for healing were free incisions of such a character as allowed the tissues to drain automatically, *e.g.* transverse incisions in dependent positions and mechanical cleaning by irrigation.

The conditions under which we worked very soon demonstrated the absurdity of trusting to a sepsis alone for prophylactic purposes. Every dressing was attended by swarms of flies, and it was much better and simpler to have towels and dressings all soaked in carbolic than to sterilise them by heat, and allow pus-soaked flies to contaminate them on every exposure.

We had a remarkable demonstration of the influence of shell fire, and soil and climatic conditions in the production of sepsis. During the winter of 1915–16 we received the wounded from the Senussi fighting. This was almost entirely an affair of rifles and machine-guns, carried on in the deserts of North Africa, and amongst these casualties it was very rare indeed ever to see a septic case, merely the clean scabbed wounds characteristic of the South African War.

In selecting certain classes of cases for special description, I am guided by what remain in my memory as those of greatest interest, importance or difficulty. Three of such there are, viz. those of wounded blood vessels, brains and bones.

*Injuries of Blood Vessels.*—First a few words about secondary hemorrhage. This to me will always remain as the nightmare of military surgery. It seemed to occur in kind of epidemics, and on certain days one had the feeling that, having dealt with one case, everything had to be kept in readiness for the next. In one week we had to ligature or amputate for hemorrhage from the brachial, sub-clavian, radial, ulnar, femoral, popliteal (twice), tibial and carotid. Space will permit me to refer to only three points in this subject.

The only satisfactory way of dealing with secondary hemorrhage is freely to open up the tissues and ligature the bleeding points, being careful to secure both the distal as well as the proximal end of the severed artery. If this could be done satisfactorily, it was very rare for any further bleeding to occur.

Proximal ligature of the main artery above the wound is most thoroughly unsatisfactory. Either it is ineffectual, or else in the case of a limb it causes gangrene, and this gangrene is of a fulminating septic character, very dangerous to life. In one case (brachial) prompt amputation saved the patient's life, but in another (femoral) the patient died of septic gangrene before removal of the limb could be effected.

In one type of case the condition is very puzzling. Here the bleeding is comparatively late, about the third or fourth week, and occurs in a case which seems to have been doing well. The bleeding occurs, not from a single large artery, but oozes up from all the tissues alike. Such a hemorrhage devitalises the patient out of all proportion to the amount of blood lost, and amputation is generally out of the question. Apart from merely cleaning and packing the wound, we found one remedy which was very useful, and that was the use of a 1 in 1,000 solution of brilliant green. This seemed to have considerable properties both of astringency and penetration.

Operations upon aneurysms were among the most exciting and the most difficult that we had to perform. We had some 14 of these cases, viz. carotid 1, subclavian 2, brachial 1, external iliac 1, femoral 3, popliteal 3, and tibial 3. It would be undesirable to go into details about these, and I will confine myself to certain general observations. The first is that it was quite exceptional to find any true aneurysmal sac, and this made any question of intra-saccular or conservative operations impossible. The next point is similar to what was said about secondary hemorrhage, viz. the futility of proximal ligature, whether done for the cure or merely as a temporary measure, preliminary to opening the aneurysm. This was especially noticeable in the subclavian cases. Ligature of the subclavian artery above the aneurysm made little or no difference, and on exposing the site of injury bleeding was terrific, and could only be finally arrested by wide exposure, digital pressure over the spouting points, and eventual ligature.

In the limbs a tourniquet above the lesion makes matters comparatively simple. It is noteworthy how very little difference the presence of a traumatic aneurysm makes to the pulse in the vessels beyond. Again and again we noted that the distal pulse was unaffected, and yet operation revealed that the main artery had been cut across. This must be due not only to the circulation going on through the open distal end of the wounded artery, but also to the efficiency of the collateral circulation, which latter circumstance also accounts for the inadequacy of a proximal ligature.

*Brains.*—We had the very great advantage of the presence of Sir Victor Horsley in our unit, originally as head of the surgical division and afterwards as Consulting Surgeon. His methods and the results attained by them are fully set out by Captain Whitaker in the April number of the *British Journal of Surgery*, who took over this especial work after Sir Victor ceased to be an active member of our staff.

There were 118 cases of gunshot cranial wounds, of which all but 16 involved the brain, *i.e.* were intra-dural. In 104 cases operation was performed for cleaning the wound and for the relief of intra-cranial pressure or suppuration. The mortality was 19 cases, a surprisingly low figure when the severity of the cases is taken into account.

Captain Whitaker's paper contains all the details of these cases, and I will merely refer to certain outstanding features of interest. First, it must be premised that this series represents only those who have survived seven to ten days after the injury, and takes no account of the many who must have died immediately or soon after the receipt of the wound. In such cases it was very remarkable how tolerant the brain was both to extensive injury and to virulent septic infection. Almost any degree of injury or sepsis seemed recoverable, except that streptococcal infection was generally fatal and ventricular injury always.

Nearly all cases of septic splintered cranial wounds require early operation both for the free drainage of the wound and the removal of pieces of bone. The main object of the operation is free removal of the cranial wall both for decompression and cleansing. Searching for foreign bodies is undesirable. The success of the operation depends largely on its thoroughness, combined with speed and accurate hemostasis. The latter was achieved by placing pressure forceps on all bleeding points in the scalp, and leaving these in place for twenty-four hours, and in using a bit of muslin to stop bleeding from vessels in the brain or its coverings.

There was a remarkable difference in the streptococcal and the staphylococcal infections. In the former, which were usually fatal, no adhesions occurred between the meninges and the skull to limit infective inflammation, and the patient died of diffuse meningitis, spreading from the wound. In the latter the wound was very early shut off by meningeal adhesions, and if a fatal result occurred, this was by an infection of the ventricles, and thence by the ependyma of the basal meninges.

Curiously enough, the pyocyaneus, which is so common an organism in bone wounds of the limbs, never once made its appearance in our series of head wounds.

The dressings of the head wounds and their drainage are matters of great importance. Tubes of all sorts were avoided. The operation wound was left widely open, and covered with a perforated sheet of rubber tissue and gauze. This was changed every twelve hours, being loosened by the application of peroxide of hydrogen.

One result of the very free removal of bone fractures by Sir Victor Horsley and Captain Whitaker was seen in the spontaneous subsidence of the cerebral hernia which followed this procedure. And it should be noted that whilst bone was very freely removed, the dura was treated with the utmost conservatism consistent with removal of bits of bone or metal which had penetrated it. The dura is not the unyielding membrane that it is usually considered. After such an operation it would allow the brain beneath to swell up considerably into the wound, and then as inflammation subsided it would collapse below the surface.

*Bones.*—The subject of the supervision of fractures was the one which I reserved to myself, in which work I was most ably assisted by Captain T. H. Brown, who before going to Egypt was for some months resident at the Southmead section of the Second Southern General Hospital. I was allowed by the War Office to take with me a mechanic, whose work it was to make such apparatus for us as we required. Soon after the rush of work had begun, it became evident that there was a very serious shortage of splints of all kinds throughout the Expeditionary Force. And as we had already begun to make these things for our own use, the authorities decided to extend the business so as to be able to supply hospital ships and other hospital units. A small disused fort, which had not been used except as a lumber room since the bombardment of Alexandria in 1880, was discovered on the outlying part of the hospital ground. This made an excellent factory, and with two Englishmen and twelve native workmen we were able to turn out a large quantity of splints. At first we made all sorts of stock patterns of wooden splints, but very quickly we found, both by our own experience and by the demands of others, that the light metal splints were much superior to anything else because of their cleanliness and durability. We obtained our material from the Ordnance Stores, and with mild steel, wire and flats we were able with quite simple tools to make quickly splints suitable for all gunshot fractures. The great advantage of this arrangement was that we had side by side the large hospital, in which hundreds of fracture cases were actually under treatment on the splints, and the factory where the splints were made and supplied, or where any modification could be made at a moment's notice. So that for inquirers, it was only a step from one place to the other ; in the one the use of the splint was a matter of practical demonstration, and in the other it was supplied ready for use.

I do not propose to describe those types of splint which we finally evolved in any detail, as I have already done so in recent publications (*Lancet*, April 29th, *British Journal of Surgery*, April). I will content myself with certain generalisations.

A splint for a gunshot fracture must be all-metal, so as not to absorb blood and pus, or to give harbour to vermin. It should take the form of a light metal frame which outlines the limb when the latter is held in a position of physiological rest with all its joints semi-flexed. It is prepared for application by making a cradle of strips of flannel or rubber bandage slung between the opposite bars of the frame upon which the wounded limb lies. It must provide for the maintenance of the broken limb in good position by the principle of extension of the broken bone in its long axis, so that no constricting or retentive bandage shall be necessary. Thus the wound is open for dressing or inspection as often as necessary, and the doing of the dressing in no way disturbs the fracture. The splint ought, if possible, to be self-contained, *i.e.* not dependent for its action or use upon attachment to any outside support. In military work constant moving of the patient must be the order of the day, and whether it is to the X-ray room or to the ship for home, it is of great importance to be able at a moment's notice to place the patient, splint and all, on to a stretcher without disturbing the alignment of his fracture.

I have added to this paper various illustrations taken from recent articles which will, with the attached legends, serve to show how for various conditions these requirements were carried out. And the fact that between October and March, five thousand, of these special splints were made and distributed is a satisfactory evidence of how they were appreciated.

As regards the actual results obtained in the treatment of gunshot fractures in our hospital, the difficulty arises that all the more simple cases were so quickly sent on to England that of these we can say nothing. The only group which remained with us sufficiently long to enable any statement to be made about results was that of the fractured femur. Of these we had 84, and I have given a detailed account of the first 60 of these in the *British Journal of Surgery*. A certain number were doomed to death before their arrival. These were generally those with diffuse sepsis and septicæmia or those with involvement of the knee-joint. Others succumbed to dysentery, and others again had to submit to amputation in order to save life. There are three conditions which usually demand this procedure. First, septic injection of the knee; second, a large dirty wound which cannot be fully laid open without hemi-section of the limb; and, thirdly, secondary hemorrhage. The mortality in our cases was 10 per cent.; another 10 per cent. lost their legs; and of the remaining 80, 74 per cent. gave perfect results as far as could be judged from the period of one to three months during which they remained under our care. The remainder were cases in which either the wounds or the fracture had remained unhealed when they were sent away from us. In other words, apart from those who lost their lives or limbs, three-fourths were restored to good functional recovery. This result was obtained chiefly by employing accurate and powerful extension by means of a transfixion pin driven through the lower end of the femur or through the head of the tibia.

